# Hybrid choice model dataset of a representative Swiss online panel survey on peoples’ preferences related to mixed renewable energy scenarios in landscapes and the effect of landscape-technology fit

**DOI:** 10.1016/j.dib.2021.107025

**Published:** 2021-04-16

**Authors:** B. Salak, K. Lindberg, F. Kienast, M. Hunziker

**Affiliations:** a Swiss Federal Institute for Forest, Snow and Landscape Research WSL, Social Sciences in Landscape Research Group, Research Unit Economics and Social Sciences, Zürcherstrasse 111, 8903 Birmensdorf, Switzerland; b Oregon State University-Cascades, Department of Forest Ecosystems and Society, 1500 SW Chandler Avenue, Bend, OR 97702, United States; c Swiss Federal Institute for Forest, Snow and Landscape Research WSL, Land Change Science Research Group, Research Unit Land-use Systems, Zürcherstrasse 111, 8903 Birmensdorf, Switzerland

**Keywords:** Place-technology fit, Landscape-technology fit, Perceived landscape quality, Landscape meanings, Renewable energy meanings, Mixed renewable energy landscapes, Hybrid choice model, Integrated choice and latent variable model

## Abstract

We present stated preference data based on a national representative Swiss online panel survey related to preference of mixed renewable energy infrastructure in landscapes. Data were collected between November 2018 and March 2019 via an online questionnaire and yielded 1026 responses. The online questionnaire consisted of two main parts – (1) questions covering meanings related to landscapes, nature and renewable energy infrastructure and questions regarding the “fit” of landscape/renewable energy infrastructure (REI) combinations and (2) a stated choice experiment. While in the first part of the questionnaire we asked respondents about their personal connection to certain landscapes, to nature and to specific REI, we also asked them to evaluate the fitting of seven different Swiss landscapes (near natural alpine areas, northern alps, touristic alpine areas, agricultural plateau, urban plateau, Jura ridges, urban alpine valley) with five different REI (wind, PV ground/agricultural, PV ground/other, PV roof, power lines) combinations. In the second part of the questionnaire, the stated choice experiment confronted respondents with 15 consecutive choice tasks, with each task involving a choice between two “energy system transformation” options and an opt-out option (none). Each choice option (beside the opt-out option) included four unlabeled attributes (landscape, wind energy infrastructure, photovoltaic energy infrastructure, high voltage overhead power line infrastructure) with varying levels. Due to data cleaning procedures (item nonresponse) the number of responses used within hybrid choice modeling and analysis was *n* = 844 (12,660 choice observations). An analysis of the hybrid choice model and further insights are presented in the article “How landscape-technology fit affects public evaluations of renewable energy infrastructure scenarios. A hybrid choice model.”

## Specifications Table

**Table:** 

Subject	Social Science
Specific subject area	Perceived landscape quality
Type of data	CSV data file
How data were acquired	Online questionnaire Sawtooth
Data format	Raw data
Parameters for data collection	The online panel survey targeted Swiss residents and is representative regarding language, gender, age, education and landscape.
Description of data collection	Data were collected with panel operator BILENDI and were administered via Sawtooth Software. Active panel members in Switzerland were invited to participate. Two reminders were sent. The questionnaire consisted of two parts, a choice experiment and questions covering meanings related to landscapes, nature and renewable energy infrastructure (REI), including the “fit” of landscape/REI combinations.
Data source location	Institution: Swiss federal research institute WSL Country: Switzerland
Data accessibility	Data is accessible via EnviDat, the WSL data portal Repository name: EnviDat (https://www.envidat.ch/) Data identification number: https://doi.org/10.16904/envidat.206. Direct URL to data: https://www.envidat.ch/dataset/landscape-technology-fit-public-evaluation
Related research article	B. Salak, K. Lindberg, F. Kienast, M. Hunziker, How landscape-technology fit affects public evaluations of renewable energy infrastructure scenarios. A hybrid choice model, Renewable and Sustainable Energy Reviews. In Press.

## Value of the Data

• Presented data provide information on public preferences across different energy scenarios. They also provide a proof-of-concept for “landscape-technology fit” and contain information about predictors (landscape- and renewable energy meanings, exposure) of peoples’ preferences related to landscape developments. Also, the dataset highlights the interconnectedness of landscape and energy aspects in terms of the perceived landscape quality and its potential relevance for decision making processes.
• The consideration of meanings for decision making processes and policy making (not only visual aspects) could be brought into all policy areas and technical decision-making tools, even those that are not landscape-oriented. During communication and planning residents of potential energy sites could be (1) informed early on and (2) invited to participatory workshops in which the meaning of landscape and REI is addressed in addition to usual visual scenarios and (3) discussing siting alternatives.
• The dataset can be used to operationalize landscape-technology fit (LTF) concept which derived from place-technology fit (PTF). In particular, this dataset may be used as a base line for future LTF model improvements in alpine regions. They contain explicit information on meanings ascribed to alpine landscapes and to specific renewable energy infrastructures.

## 1 Data Description

We conducted a representative online panel survey in Switzerland between November 2018 and March 2019 to elicit the preferences of Swiss residents for landscape oriented renewable energy infrastructure developments. The questionnaire was developed by WSL and operated by panel provider BILENDI GmbH. The survey is representative in language, age, gender, education and landscape.

**Fig. 1:**
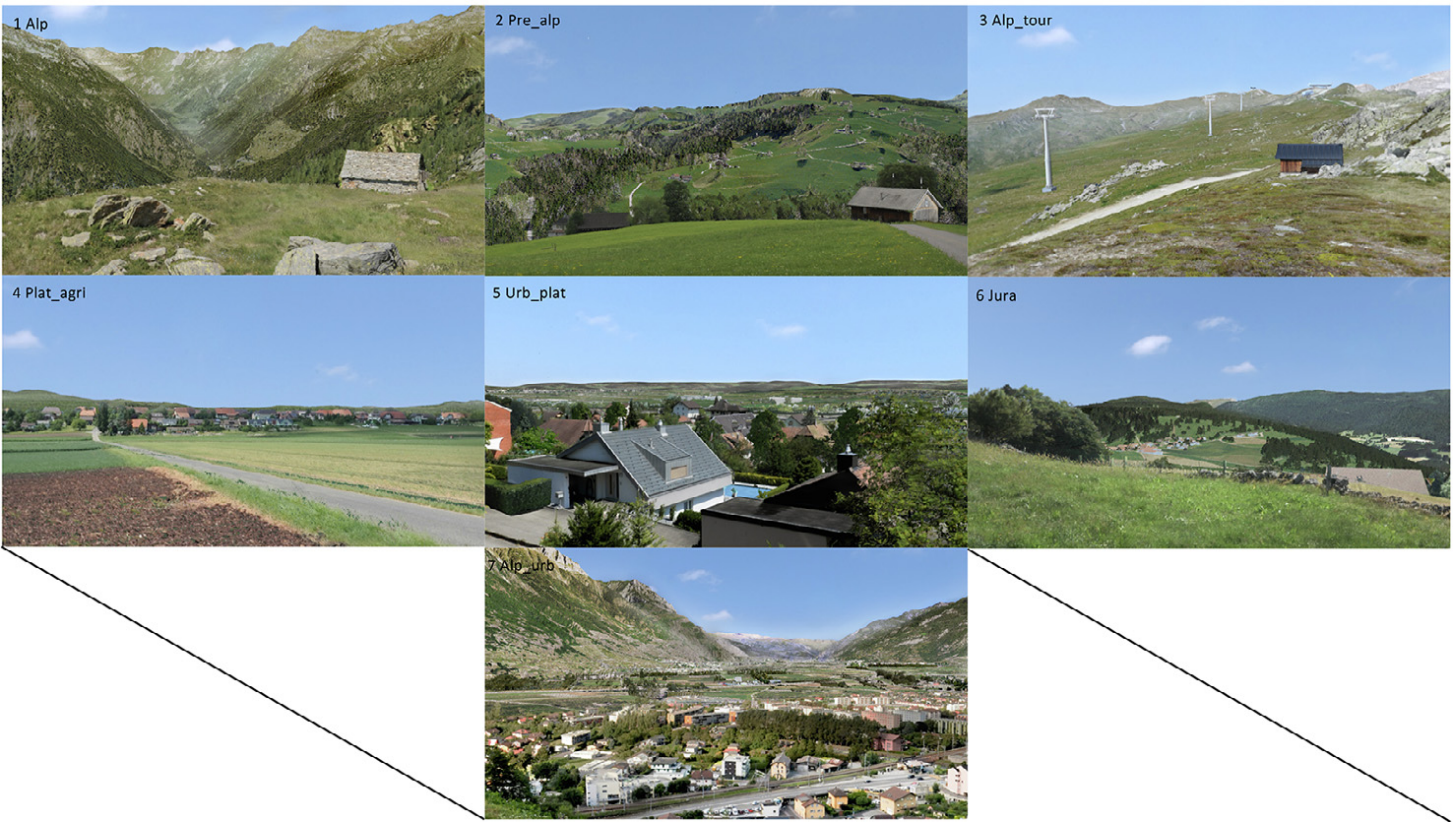
Landscape visualizations used in this study.

**Fig. 2:**
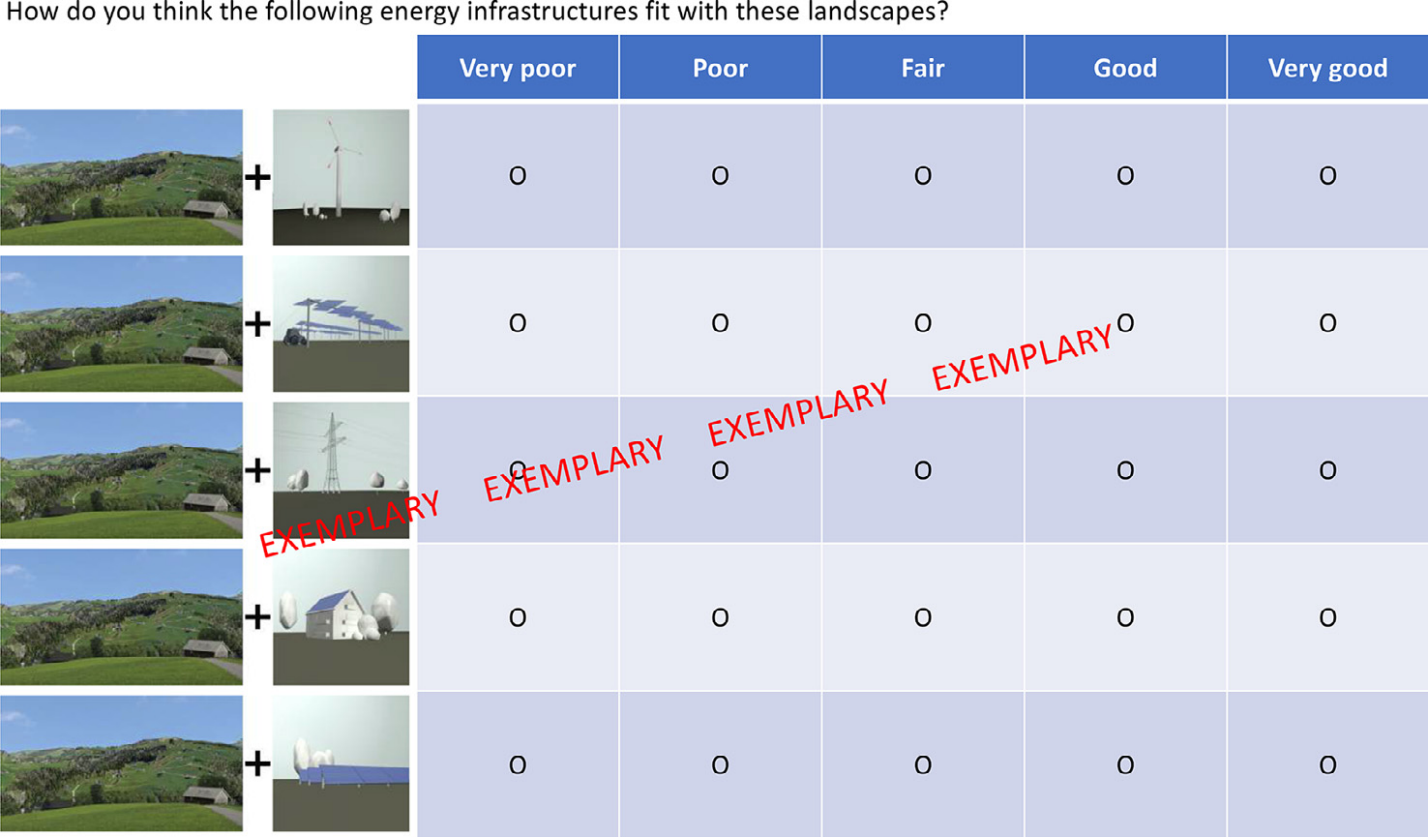
Exemplary set of landscape-technology fit evaluation.

The questionnaire consisted of two major parts, where within the first part questions were related (1) to meanings ascribed to landscapes and renewable energy infrastructure, (2) to aspects of landscape-technology fit and (3) to exposure of people to landscapes and renewable energy infrastructures. Within the second part a stated choice model was presented. All respondents were designated to one of seven landscapes (near natural alpine areas, northern alps, touristic alpine areas, agricultural plateau, urban plateau, jura ridges, urban alpine valley) according to the ZIP code of their origin. The landscape visualizations used in this study are illustrated in Fig. 1, whereas further details about its joint development can be found in Spielhofer et al. [1]. All survey items and scales are presented in Table 1, whereas the questionnaire is added to the supplementary material of the present artice. Socio demographic items and respondent ID were provided by the panel provider (items 1 to 6). After starting the survey, respondents were first asked to select landscapes that most closely represent the landscape of their living, recreation and childhood environment (variables 160–162). In a next step, respondents were asked to evaluate (randomly presented) meanings ascribed to each of the seven landscapes presented. A generalized overview of the evaluation of landscape meaning items (variables 84 to 153) is provided in Table 2. Consequently, respondents were asked about (randomly presented) meanings they ascribe to each of three renewable energy infrastructures (wind, PV ground, PV roof). A descriptive overview is provided in Table 3 (variables 57 to 83). As a consequence, people were asked to evaluate their personal feeling of the “fit” of each landscape/renewable energy infrastructure combination (variables 22 to 56). Within this landscape-technology fit evaluation photovoltaic infrastructure was separated into open space ground mounted PV and agricultural PV infrastructure. In addition, high voltage overhead power lines were integrated. For the evaluation, the landscape/energy infrastructure combination for each landscape was randomized in appearance. An exemplary illustration of the operationalized landscape-technology fit concept can be found in Fig. 2, while an overview of respondents evaluation can be found in Table 4. Lastly, people were asked about how they would feel if they would be exposed to renewable energy infrastructure in their living (items 154 to 156) and their recreation environment (items 157 to 159).

**Table 1:** Item based description of the dataset.

Var_num	Var_code	Var_descr	1	2	3	4	5	6	7
1	sys_RespNum	Respondent ID	–						
2	Lang	Language region	Swiss-German	Swiss-French	Swiss-Italian				
3	Gend	Gender	Female	Male					
4	Age	Age	18–24	25–34	35–44	45–54	55–64		
5	Edu	Education	obligatory school	secondary level: professional education	secondary level: general education	tertiar level: professional education	tertiar level: universities		
6	Ls	ZIP designated Landscape	Alp	Northern prealps	Touristic alpine areas	Agricultural Plateau	Urban plateau	Jura ridges	Urban alpine valley
7–21	CE1_Random1–15	Random Choice task 1	–						
22–56	LTFaband-alpval_r1	How do you think the following energy infrastructures fit with these landscapes? (LS1–7+Powerlines)	very poor	poor	fair	good	very good		
23	LTFaband-alpval_r2	How do you think the following energy infrastructures fit with these landscapes? (LS1–7+PVagri)	very poor	poor	fair	good	very good		
24	LTFaband-alpval_r3	How do you think the following energy infrastructures fit with these landscapes? (LS1–7+PVground)	very poor	poor	fair	good	very good		
25	LTFaband-alpval_r4	How do you think the following energy infrastructures fit with these landscapes? (LS1–7+PVroof)	very poor	poor	fair	good	very good		
26	LTFaband-alpval_r5	How do you think the following energy infrastructures fit with these landscapes? (LS1–7+Wind)	very poor	poor	fair	good	very good		
27	LTFprealps_r1	How do you think the following energy infrastructures fit with these landscapes? (Pre_alps+Powerlines)	very poor	poor	fair	good	very good		
28	LTFprealps_r2	How do you think the following energy infrastructures fit with these landscapes? (Pre_alps+PVagri)	very poor	poor	fair	good	very good		
29	LTFprealps_r3	How do you think the following energy infrastructures fit with these landscapes? (Pre_alps+PVground)	very poor	poor	fair	good	very good		
30	LTFprealps_r4	How do you think the following energy infrastructures fit with these landscapes? (Pre_alps+PVroof)	very poor	poor	fair	good	very good		
31	LTFprealps_r5	How do you think the following energy infrastructures fit with these landscapes? (Pre_alps+Wind)	very poor	poor	fair	good	very good		
32	LTFalptour_r1	How do you think the following energy infrastructures fit with these landscapes? (Alp_tour+Powerlines)	very poor	poor	fair	good	very good		
33	LTFalptour_r2	How do you think the following energy infrastructures fit with these landscapes? (Alp_tour+PVagri)	very poor	poor	fair	good	very good		
34	LTFalptour_r3	How do you think the following energy infrastructures fit with these landscapes? (Alp_tour+PVground)	very poor	poor	fair	good	very good		
35	LTFalptour_r4	How do you think the following energy infrastructures fit with these landscapes? (Alp_tour+PVroof)	very poor	poor	fair	good	very good		
36	LTFalptour_r5	How do you think the following energy infrastructures fit with these landscapes? (Alp_tour+Wind)	very poor	poor	fair	good	very good		
37	LTFplatagri_r1	How do you think the following energy infrastructures fit with these landscapes? (Plat_agri+Powerlines)	very poor	poor	fair	good	very good		
38	LTFplatagri_r2	How do you think the following energy infrastructures fit with these landscapes? (Plat_agri+PVagri)	very poor	poor	fair	good	very good		
39	LTFplatagri_r3	How do you think the following energy infrastructures fit with these landscapes? (Plat_agri+PVground)	very poor	poor	fair	good	very good		
40	LTFplatagri_r4	How do you think the following energy infrastructures fit with these landscapes? (Plat_agri+PVroof)	very poor	poor	fair	good	very good		
41	LTFplatagri_r5	How do you think the following energy infrastructures fit with these landscapes? (Plat_agri+Wind)	very poor	poor	fair	good	very good		
42	LTFplaturb_r1	How do you think the following energy infrastructures fit with these landscapes? (Plat_urb+Powerlines)	very poor	poor	fair	good	very good		
43	LTFplaturb_r2	How do you think the following energy infrastructures fit with these landscapes? (Plat_urb+PVagri)	very poor	poor	fair	good	very good		
44	LTFplaturb_r3	How do you think the following energy infrastructures fit with these landscapes? (Plat_urb+PVground)	very poor	poor	fair	good	very good		
45	LTFplaturb_r4	How do you think the following energy infrastructures fit with these landscapes? (Plat_urb+PVroof)	very poor	poor	fair	good	very good		
46	LTFplaturb_r5	How do you think the following energy infrastructures fit with these landscapes? (Plat_urb+Wind)	very poor	poor	fair	good	very good		
47	LTFjura_r1	How do you think the following energy infrastructures fit with these landscapes? (Jura+Powerlines)	very poor	poor	fair	good	very good		
48	LTFjura_r2	How do you think the following energy infrastructures fit with these landscapes? (Jura+PVagri)	very poor	poor	fair	good	very good		
49	LTFjura_r3	How do you think the following energy infrastructures fit with these landscapes? (Jura+PVground)	very poor	poor	fair	good	very good		
50	LTFjura_r4	How do you think the following energy infrastructures fit with these landscapes? (Jura+PVroof)	very poor	poor	fair	good	very good		
51	LTFjura_r5	How do you think the following energy infrastructures fit with these landscapes? (Jura+Wind)	very poor	poor	fair	good	very good		
52	LTFalpval_r1	How do you think the following energy infrastructures fit with these landscapes? (Alp_urb+Powerlines)	very poor	poor	fair	good	very good		
53	LTFalpval_r2	How do you think the following energy infrastructures fit with these landscapes? (Alp_urb+PVagri)	very poor	poor	fair	good	very good		
54	LTFalpval_r3	How do you think the following energy infrastructures fit with these landscapes? (Alp_urb+PVground)	very poor	poor	fair	good	very good		
55	LTFalpval_r4	How do you think the following energy infrastructures fit with these landscapes? (Alp_urb+PVroof)	very poor	poor	fair	good	very good		
56	LTFalpval_r5	How do you think the following energy infrastructures fit with these landscapes? (Alp_urb+Wind)	very poor	poor	fair	good	very good		
57	REwind_r1	Wind energy infrastructure provides clean energy	strongly disagree	disagree	in between	agree	strongly agree		
58	REwind_r2	Wind energy infrastructure secures jobs	strongly disagree	disagree	in between	agree	strongly agree		
59	REwind_r3	Wind energy infrastructure supports local economy	strongly disagree	disagree	in between	agree	strongly agree		
60	REwind_r4	Wind energy infrastructure cannot replace other energy sources in CH	strongly disagree	disagree	in between	agree	strongly agree		
61	REwind_r5	Wind energy infrastructure deliver limited yield	strongly disagree	disagree	in between	agree	strongly agree		
62	REwind_r7	Wind energy infrastructure ensures variety in the landscape	strongly disagree	disagree	in between	agree	strongly agree		
63	REwind_r9	Wind energy infrastructure represent the progress of humans	strongly disagree	disagree	in between	agree	strongly agree		
64	REwind_r12	Wind energy infrastructure contribute to solving the most important problems of humanity	strongly disagree	disagree	in between	agree	strongly agree		
65	REwind_r13	Wind energy infrastructure represent awakening	strongly disagree	disagree	in between	agree	strongly agree		
66	REpvground_r1	PV ground infrastructure provides clean energy	strongly disagree	disagree	in between	agree	strongly agree		
67	REpvground_r2	PV ground infrastructure secures jobs	strongly disagree	disagree	in between	agree	strongly agree		
68	REpvground_r3	PV ground infrastructure supports local economy	strongly disagree	disagree	in between	agree	strongly agree		
69	REpvground_r4	PV ground infrastructure cannot replace other energy sources in CH	strongly disagree	disagree	in between	agree	strongly agree		
70	REpvground_r5	PV ground infrastructure deliver limited yield	strongly disagree	disagree	in between	agree	strongly agree		
71	REpvground_r7	PV ground infrastructure ensures variety in the landscape	strongly disagree	disagree	in between	agree	strongly agree		
72	REpvground_r9	PV ground infrastructure represent the progress of humans	strongly disagree	disagree	in between	agree	strongly agree		
73	REpvground_r12	PV ground infrastructure contribute to solving the most important problems of humanity	strongly disagree	disagree	in between	agree	strongly agree		
74	REpvground_r13	PV ground infrastructure represent awakening	strongly disagree	disagree	in between	agree	strongly agree		
75	REpvroof_r1	PV roof infrastructure provides clean energy	strongly disagree	disagree	in between	agree	strongly agree		
76	REpvroof_r2	PV roof infrastructure secures jobs	strongly disagree	disagree	in between	agree	strongly agree		
77	REpvroof_r3	PV roof infrastructure supports local economy	strongly disagree	disagree	in between	agree	strongly agree		
78	REpvroof_r4	PV roof infrastructure cannot replace other energy sources in CH	strongly disagree	disagree	in between	agree	strongly agree		
79	REpvroof_r5	PV roof infrastructure deliver limited yield	strongly disagree	disagree	in between	agree	strongly agree		
80	REpvroof_r7	PV roof infrastructure ensures variety in the landscape	strongly disagree	disagree	in between	agree	strongly agree		
81	REpvroof_r9	PV roof infrastructure represent the progress of humans	strongly disagree	disagree	in between	agree	strongly agree		
82	REpvroof_r12	PV roof infrastructure contribute to solving the most important problems of humanity	strongly disagree	disagree	in between	agree	strongly agree		
83	REpvroof_r13	PV roof infrastructure represent awakening	strongly disagree	disagree	in between	agree	strongly agree		
84	meaningsABAND_r1	Near natural alpine landscapes are a symbol for human progress	strongly disagree	disagree	in between	agree	strongly agree		
85	meaningsABAND_r3	Near natural alpine landscapes represent the dominance of humans over nature	strongly disagree	disagree	in between	agree	strongly agree		
86	meaningsABAND_r5	Near natural alpine landscapes represent scenic beauty	strongly disagree	disagree	in between	agree	strongly agree		
87	meaningsABAND_r6	Near natural alpine landscapes offer sense of intimicy/familiarity	strongly disagree	disagree	in between	agree	strongly agree		
88	meaningsABAND_r7	Near natural alpine landscapes help to recognize sense	strongly disagree	disagree	in between	agree	strongly agree		
89	meaningsABAND_r9	Near natural alpine landscapes help to can relax my soul	strongly disagree	disagree	in between	agree	strongly agree		
90	meaningsABAND_r10	Near natural alpine landscapes make me feeling comfortable	strongly disagree	disagree	in between	agree	strongly agree		
91	meaningsABAND_r11	Near natural alpine landscapes are a symbol for an authentic landscape	strongly disagree	disagree	in between	agree	strongly agree		
92	meaningsABAND_r12	Near natural alpine landscapes represent an intact world	strongly disagree	disagree	in between	agree	strongly agree		
93	meaningsABAND_r13	Near natural alpine landscapes help to experience myself	strongly disagree	disagree	in between	agree	strongly agree		
94	meaningsPREALPS_r1	Northern alpine landscapes are a symbol for human progress	strongly disagree	disagree	in between	agree	strongly agree		
95	meaningsPREALPS_r3	Northern alpine landscapes represent the dominance of humans over nature	strongly disagree	disagree	in between	agree	strongly agree		
96	meaningsPREALPS_r5	Northern alpine landscapes represent scenic beauty	strongly disagree	disagree	in between	agree	strongly agree		
97	meaningsPREALPS_r6	Northern alpine landscapes offer sense of intimicy/familiarity	strongly disagree	disagree	in between	agree	strongly agree		
98	meaningsPREALPS_r7	Northern alpine landscapes help to recognize sense	strongly disagree	disagree	in between	agree	strongly agree		
99	meaningsPREALPS_r9	Northern alpine landscapes help to can relax my soul	strongly disagree	disagree	in between	agree	strongly agree		
100	meaningsPREALPS_r10	Northern alpine landscapes make me feeling comfortable	strongly disagree	disagree	in between	agree	strongly agree		
101	meaningsPREALPS_r11	Northern alpine landscapes are a symbol for an authentic landscape	strongly disagree	disagree	in between	agree	strongly agree		
102	meaningsPREALPS_r12	Northern alpine landscapes represent an intact world	strongly disagree	disagree	in between	agree	strongly agree		
103	meaningsPREALPS_r13	Northern alpine landscapes help to experience myself	strongly disagree	disagree	in between	agree	strongly agree		
104	meaningsALPTOUR_r1	Alpine touristic landscapes are a symbol for human progress	strongly disagree	disagree	in between	agree	strongly agree		
105	meaningsALPTOUR_r3	Alpine touristic landscapes represent the dominance of humans over nature	strongly disagree	disagree	in between	agree	strongly agree		
106	meaningsALPTOUR_r5	Alpine touristic landscapes represent scenic beauty	strongly disagree	disagree	in between	agree	strongly agree		
107	meaningsALPTOUR_r6	Alpine touristic landscapes offer sense of intimicy/familiarity	strongly disagree	disagree	in between	agree	strongly agree		
108	meaningsALPTOUR_r7	Alpine touristic landscapes help to recognize sense	strongly disagree	disagree	in between	agree	strongly agree		
109	meaningsALPTOUR_r9	Alpine touristic landscapes help to can relax my soul	strongly disagree	disagree	in between	agree	strongly agree		
110	meaningsALPTOUR_r10	Alpine touristic landscapes make me feeling comfortable	strongly disagree	disagree	in between	agree	strongly agree		
111	meaningsALPTOUR_r11	Alpine touristic landscapes are a symbol for an authentic landscape	strongly disagree	disagree	in between	agree	strongly agree		
112	meaningsALPTOUR_r12	Alpine touristic landscapes represent an intact world	strongly disagree	disagree	in between	agree	strongly agree		
113	meaningsALPTOUR_r13	Alpine touristic landscapes help to experience myself	strongly disagree	disagree	in between	agree	strongly agree		
114	meaningsPLATAGRI_r1	Agricultural plateau landscapes are a symbol for human progress	strongly disagree	disagree	in between	agree	strongly agree		
115	meaningsPLATAGRI_r3	Agricultural plateau landscapes represent the dominance of humans over nature	strongly disagree	disagree	in between	agree	strongly agree		
116	meaningsPLATAGRI_r5	Agricultural plateau landscapes represent scenic beauty	strongly disagree	disagree	in between	agree	strongly agree		
117	meaningsPLATAGRI_r6	Agricultural plateau landscapes offer sense of intimicy/familiarity	strongly disagree	disagree	in between	agree	strongly agree		
118	meaningsPLATAGRI_r7	Agricultural plateau landscapes help to recognize sense	strongly disagree	disagree	in between	agree	strongly agree		
119	meaningsPLATAGRI_r9	Agricultural plateau landscapes help to can relax my soul	strongly disagree	disagree	in between	agree	strongly agree		
120	meaningsPLATAGRI_r10	Agricultural plateau landscapes make me feeling comfortable	strongly disagree	disagree	in between	agree	strongly agree		
121	meaningsPLATAGRI_r11	Agricultural plateau landscapes are a symbol for an authentic landscape	strongly disagree	disagree	in between	agree	strongly agree		
122	meaningsPLATAGRI_r12	Agricultural plateau landscapes represent an intact world	strongly disagree	disagree	in between	agree	strongly agree		
123	meaningsPLATAGRI_r13	Agricultural plateau landscapes help to experience myself	strongly disagree	disagree	in between	agree	strongly agree		
124	meaningsPLATURB_r1	Landscapes on the urban plateau are a symbol for human progress	strongly disagree	disagree	in between	agree	strongly agree		
125	meaningsPLATURB_r3	Landscapes on the urban plateau represent the dominance of humans over nature	strongly disagree	disagree	in between	agree	strongly agree		
126	meaningsPLATURB_r5	Landscapes on the urban plateau represent scenic beauty	strongly disagree	disagree	in between	agree	strongly agree		
127	meaningsPLATURB_r6	Landscapes on the urban plateau offer sense of intimicy/familiarity	strongly disagree	disagree	in between	agree	strongly agree		
128	meaningsPLATURB_r7	Landscapes on the urban plateau help to recognize sense	strongly disagree	disagree	in between	agree	strongly agree		
129	meaningsPLATURB_r9	Landscapes on the urban plateau help to can relax my soul	strongly disagree	disagree	in between	agree	strongly agree		
130	meaningsPLATURB_r10	Landscapes on the urban plateau make me feeling comfortable	strongly disagree	disagree	in between	agree	strongly agree		
131	meaningsPLATURB_r11	Landscapes on the urban plateau are a symbol for an authentic landscape	strongly disagree	disagree	in between	agree	strongly agree		
132	meaningsPLATURB_r12	Landscapes on the urban plateau represent an intact world	strongly disagree	disagree	in between	agree	strongly agree		
133	meaningsPLATURB_r13	Landscapes on the urban plateau help to experience myself	strongly disagree	disagree	in between	agree	strongly agree		
134	meaningsJURA_r1	Jura landscapes are a symbol for human progress	strongly disagree	disagree	in between	agree	strongly agree		
135	meaningsJURA_r3	Jura landscapes represent the dominance of humans over nature	strongly disagree	disagree	in between	agree	strongly agree		
136	meaningsJURA_r5	Jura landscapes represent scenic beauty	strongly disagree	disagree	in between	agree	strongly agree		
137	meaningsJURA_r6	Jura landscapes offer sense of intimicy/familiarity	strongly disagree	disagree	in between	agree	strongly agree		
138	meaningsJURA_r7	Jura landscapes help to recognize sense	strongly disagree	disagree	in between	agree	strongly agree		
139	meaningsJURA_r9	Jura landscapes help to can relax my soul	strongly disagree	disagree	in between	agree	strongly agree		
140	meaningsJURA_r10	Jura landscapes make me feeling comfortable	strongly disagree	disagree	in between	agree	strongly agree		
141	meaningsJURA_r11	Jura landscapes are a symbol for an authentic landscape	strongly disagree	disagree	in between	agree	strongly agree		
142	meaningsJURA_r12	Jura landscapes represent an intact world	strongly disagree	disagree	in between	agree	strongly agree		
143	meaningsJURA_r13	Jura landscapes help to experience myself	strongly disagree	disagree	in between	agree	strongly agree		
144	meaningsALPVAL_r1	Landscapes in urban alpine valleys are a symbol for human progress	strongly disagree	disagree	in between	agree	strongly agree		
145	meaningsALPVAL_r3	Landscapes in urban alpine valleys represent the dominance of humans over nature	strongly disagree	disagree	in between	agree	strongly agree		
146	meaningsALPVAL_r5	Landscapes in urban alpine valleys represent scenic beauty	strongly disagree	disagree	in between	agree	strongly agree		
147	meaningsALPVAL_r6	Landscapes in urban alpine valleys offer sense of intimicy/familiarity	strongly disagree	disagree	in between	agree	strongly agree		
148	meaningsALPVAL_r7	Landscapes in urban alpine valleys help to recognize sense	strongly disagree	disagree	in between	agree	strongly agree		
149	meaningsALPVAL_r9	Landscapes in urban alpine valleys help to can relax my soul	strongly disagree	disagree	in between	agree	strongly agree		
150	meaningsALPVAL_r10	Landscapes in urban alpine valleys make me feeling comfortable	strongly disagree	disagree	in between	agree	strongly agree		
151	meaningsALPVAL_r11	Landscapes in urban alpine valleys are a symbol for an authentic landscape	strongly disagree	disagree	in between	agree	strongly agree		
152	meaningsALPVAL_r12	Landscapes in urban alpine valleys represent an intact world	strongly disagree	disagree	in between	agree	strongly agree		
153	meaningsALPVAL_r13	Landscapes in urban alpine valleys help to experience myself	strongly disagree	disagree	in between	agree	strongly agree		
154	WBTR3_r1	Wind energy infrastructures in my living environment…	are very disturbing	are disturbing	rather disturb	neither	rather like	like	like it very much
155	WBTR3_r2	Roof mounted PV in my living environment…	are very disturbing	are disturbing	rather disturb	neither	rather like	like	like it very much
156	WBTR3_r3	Open space mounted PV in my living environment…	are very disturbing	are disturbing	rather disturb	neither	rather like	like	like it very much
157	LBTR3_r1	Wind energy infrastructures in my recreation environment…	are very disturbing	are disturbing	rather disturb	neither	rather like	like	like it very much
158	LBTR3_r2	Roof mounted PV in my recreation environment…	are very disturbing	are disturbing	rather disturb	neither	rather like	like	like it very much
159	LBTR3_r3	Open space mounted PV in my recreation environment…	are very disturbing	are disturbing	rather disturb	neither	rather like	like	like it very much
160	WumgSEL	Which of the following typical Swiss landscapes most closely represents the landscape of your living environment?	Alp	Northern prealps	Touristic alpine areas	Agricultural Plateau	Urban plateau	Jura ridges	Urban alpine valley
161	LumgSEL	Which of the following typical Swiss landscapes most closely represents the landscape of your recreation environment?	Alp	Northern prealps	Touristic alpine areas	Agricultural Plateau	Urban plateau	Jura ridges	Urban alpine valley
162	WgeschKID	Which of the following typical Swiss landscapes most closely represents the landscape of your childhood?	Alp	Northern prealps	Touristic alpine areas	Agricultural Plateau	Urban plateau	Jura ridges	Urban alpine valley

**Table 2:** Description of variables related to meanings ascribed to landscapes.

This landscape…		Response distribution (number, percentage)					Item descriptives	
Variable	Description	Strongly disagree	disagree	in between	agree	Strongly agree	Mean	SD
**Arcadian landscape perception**								
LSM_scenic-beauty	…represents scenic beauty.	1135 (9.0%)	2127 (16.8%)	2803 (22.1%)	4082 (32.2%)	2513 (19.9%)	3.37	1.23
LSM_intimicy	…offers sense of intimicy/familiarity.	852 (6.7%)	1862 (14.7%)	3340 (26.4%)	4696 (37.1%)	1910 (15.1%)	3.39	1.11
LSM_sense	…helps to recognize sense.	576 (4.5%)	1436 (11.3%)	3513 (27.8%)	5184 (40.9%)	1951 (15.4%)	3.51	1.03
LSM_relax	…helps to can relax my soul.	916 (7.2%)	2064 (16.3%)	2845 (22.5%)	4520 (35.7%)	2315 (18.3%)	3.42	1.17
LSM_comfortable	…makes me feeling comfortable.	594 (4.7%)	1619 (12.8%)	3104 (24.5%)	4983 (39.4%)	2360 (18.6%)	3.54	1.08
LSM_authenticity	…is a symbol for an authentic landscape.	707 (5.6%)	1709 (13.5%)	3228 (25.5%)	4934 (39.0%)	2082 (16.4%)	3.47	1.09
LSM_intact-world	…represents an intact world.	1170 (9.2%)	2176 (17.2%)	3066 (24.2%)	4169 (32.9%)	2079 (16.4%)	3.30	1.20
LSM_self-experience	…helps to experience myself.	892 (7.0%)	2049 (16.2%)	3666 (29.0%)	4139 (32.7%)	1914 (15.1%)	3.33	1.13
**Utilitarian landscape perception**								
LSM_progress	…is a symbol for human progress.	1313 (10.4%)	2507 (19.8%)	3982 (31.4%)	3762 (29.7%)	1096 (8.7%)	3.06	1.12
LSM_dominance	…represents the dominance of humans over nature.	1687 (13.3%)	2711 (21.4%)	3100 (24.5%)	3671 (29.0%)	1491 (11.8%)	3.04	1.23

LSM = Landscape meaning, SD = standard deviation, *N* = 12,660 choice observations.

**Table 3:** Description of items related to meanings ascribed to renewable energy infrastructure.

							Item	
		Response distribution (number, percentage)					descriptives	
Variable	Description	Strongly disagree	disagree	in between	agree	Strongly agree	Mean	SD
**Meanings ascribed to wind energy infrastructure.**								
**Perceived contribution to sustainability**								
Wind_clean_energy	…provide clean energy.	120 (0.9%)	375 (3.0%)	1800 (14.2%)	6435 (50.8%)	3930 (31.0%)	4.08	0.81
Wind_create_jobs	…potential to create jobs.	405 (3.2%)	1245 (9.8%)	3510 (27.7%)	5655 (44.7%)	1845 (14.6%)	3.58	0.96
Wind_support_local_economy	…support local economy.	270 (2.1%)	960 (7.6%)	4155 (32.8%)	5760 (45.5%)	1515 (12.0%)	3.58	0.87
Wind_progress_humans	…represent the progress of humans.	435 (3.4%)	900 (7.1%)	3210 (25.4%)	6255 (49.4%)	1860 (14.7%)	3.65	0.93
Wind_solving_problems	…contribute to solving the most important problems of humanity.	870 (6.9%)	1635 (12.9%)	3735 (29.5%)	4845 (38.3%)	1575 (12.4%)	3.36	1.07
Wind_awakening	…represent awakening.	525 (4.2%)	1140 (9.0%)	3525 (27.8%)	5415 (42.8%)	2055 (16.2%)	3.58	1.00
**Perceived contribution to a mechanized world**								
Wind_no_replacement	…cannot replace other energy sources in Switzerland.	945 (7.5%)	3135 (24.8%)	3690 (29.1%)	3660 (28.9%)	1230 (9.7%)	3.09	1.10
Wind_limited_yield	…deliver limited yield.	420 (3.3%)	1875 (14.8%)	3825 (30.2%)	5250 (41.5%)	1290 (10.2%)	3.40	0.97
Wind_distract	…distract from really important measures.	1305 (10.3%)	3315 (26.2%)	4365 (34.5%)	2850 (22.5%)	825 (6.5%)	2.89	1.07
**Meanings ascribed to ground-mounted PV infrastructures.**								
**Perceived contribution to sustainability**								
PVground_clean_energy	…provide clean energy.	225 (1.8%)	615 (4.9%)	2010 (15.9%)	6345 (50.1%)	3465 (27.4%)	3.96	0.89
PVground_create_jobs	…potential to create jobs	285 (2.2%)	990 (7.8%)	3060 (24.2%)	6315 (49.9%)	2010 (15.9%)	3.69	0.91
PVground_support_local_economy	…support local economy.	225 (1.8%)	780 (6.2%)	3615 (28.5%)	6315 (49.9%)	1725 (13.6%)	3.67	0.85
PVground_progress_humans	…represent the progress of humans.	255 (2.0%)	885 (7.0%)	2835 (22.4%)	6570 (51.9%)	2115 (16.7%)	3.74	0.89
PVground_solving_problems	…contribute to solving the most important problems of humanity.	660 (5.2%)	1440 (11.4%)	3765 (29.7%)	5310 (41.9%)	1485 (11.7%)	3.44	1.01
PVground_awakening	…represent awakening.	390 (3.1%)	975 (7.7%)	3645 (28.8%)	5730 (45.3%)	1920 (15.2%)	3.62	0.94
**Perceived contribution to a mechanized world**								
PVground_no_replacement	…cannot replace other energy sources in Switzerland.	1035 (8.2%)	3315 (26.2%)	3720 (29.4%)	3585 (28.3%)	1005 (7.9%)	3.02	1.09
PVground_limited_yield	…deliver limited yield.	525 (4.2%)	2175 (17.2%)	4185 (33.1%)	4740 (37.4%)	1035 (8.2%)	3.28	0.98
PVground_distract	…distract from really important measures.	1335 (10.5%)	3045 (24.1%)	4560 (36.0%)	3030 (23.9%)	690 (5.5%)	2.90	1.05
**Meanings ascribed to roof-mounted PV infrastructures.**								
**Perceived contribution to sustainability**								
PVroof_clean_energy	…provide clean energy.	180 (1.4%)	420 (3.3%)	1875 (14.8%)	5820 (46.0%)	4365 (34.5%)	4.09	0.86
PVroof_create_jobs	…potential to create jobs	225 (1.8%)	1050 (8.3%)	2790 (22.0%)	6240 (49.3%)	2355 (18.6%)	3.75	0.91
PVroof_support_local_economy	…support local economy.	210 (1.7%)	645 (5.1%)	3090 (24.4%)	6225 (49.2%)	2490 (19.7%)	3.80	0.87
PVroof_progress_humans	…represent the progress of humans.	210 (1.7%)	360 (2.8%)	2010 (15.9%)	6750 (53.3%)	3330 (26.3%)	4.00	0.83
PVroof_solving_problems	…contribute to solving the most important problems of humanity.	450 (3.5%)	1080 (8.5%)	3480 (27.5%)	5535 (43.7%)	2115 (16.7%)	3.61	0.98
PVroof_awakening	…represent awakening.	195 (1.5%)	555 (4.4%)	2565 (20.3%)	6420 (50.7%)	2925 (23.1%)	3.89	0.86
**Perceived contribution to a mechanized world**								
PVroof_no_replacement	…cannot replace other energy sources in Switzerland.	1230 (9.7%)	3480 (27.5%)	3345 (26.4%)	3480 (27.5%)	1125 (8.9%)	2.98	1.14
PVroof_limited_yield	…deliver limited yield.	510 (4.0%)	2340 (18.5%)	4125 (32.6%)	4560 (36.0%)	1125 (8.9%)	3.27	0.99
PVroof_distract	…distract from really important measures.	1785 (14.1%)	3570 (28.2%)	3900 (30.8%)	2610 (20.6%)	795 (6.3%)	2.77	1.12

*Note:* SD = standard deviation, *N* = 12,660 choice observations.

**Table 4:** Description of items related to landscape-technology fit.

							Item	
Perceived fit of…		Response distribution (number, percentage)					descriptives	
Variable	Description	very poor	poor	fair	good	very good	Mean	SD
LTF_Wind	…wind energy infrastructure to presented landscape.	1876 (14.8%)	2146 (17.0%)	3044 (24.0%)	3542 (28.0%)	2052 (16.2%)	3.14	1.29
LTF_PVagri^a^	…PV-infrastructure mounted on agricultural land to presented landscape.	2394 (18.9%)	2909 (23.0%)	3154 (24.9%)	2828 (22.3%)	1375 (10.9%)	2.83	1.27
LTF_PVground^a^	…PV-infrastructure mounted on other land to presented landscape.	2102 (16.6%)	2517 (19.9%)	3354 (26.5%)	3255 (25.7%)	1432 (11.3%)	2.95	1.25
LTF_PVroof	…PV-infrastructure mounted on roofs to presented landscape.	832 (6.6%)	1037 (8.2%)	1864 (14.7%)	3426 (27.1%)	5501 (43.5%)	3.93	1.22
LTF_Power-line	…power line infrastructure to presented landscape.	3160 (25.0%)	2821 (22.3%)	3301 (26.1%)	2394 (18.9%)	984 (7.8%)	2.62	1.26
*Note:*								

SD = standard deviation, LTF = Landscape-technology fit, *N* = 12,660 choice observations.

a The mean of these two variables was used to create a new variable reflecting ground-based PV infrastructure.

The second part of the online panel survey consisted of a discrete choice study in which respondents faced 15 consecutive choice tasks. Respondents were asked to choose among two landscape oriented renewable energy infrastructure alternatives and one opt-out option. Each of these alternatives (beside the opt-out option) had four attributes (landscape, wind energy infrastructure, PV infrastructure, power line infrastructure). Choice design, consecutive choice tasks and choice attributes are presented in Table 5. An exemplary choice task is illustrated in Salak et al. [2].

**Table 5:** Description of choice tasks, choice attributes and attribute levels.

Choice Task	Landscape	Wind	PV	PL	Landscape	Wind	PV	PL	opt out possibility
1	1	1	1	2	1	3	2	1	Yes
2	7	1	1	2	5	2	3	1	Yes
3	1	3	2	2	6	4	1	2	Yes
4	7	4	3	1	6	3	2	1	Yes
5	5	4	4	1	2	2	3	1	Yes
6	4	2	2	1	2	1	4	2	Yes
7	6	3	3	1	3	4	2	2	Yes
8	2	4	4	1	5	2	4	2	Yes
9	3	2	4	1	4	1	3	2	Yes
10	2	2	1	2	3	3	3	2	Yes
11	6	2	3	2	1	1	4	1	Yes
12	7	3	1	2	7	2	4	2	Yes
13	5	4	2	2	4	4	1	1	Yes
14	4	3	4	1	7	4	1	1	Yes
15	3	1	3	2	7	3	2	2	Yes

Choice	1				2				3
						

Attribute Landscape					Attriute Wind energy infrastructure				

1	Alp	Near natural alpine areas			1	No Wind energy infrastructure			
2	Pre_alp	Northern prealps			2	Low Level of wind infrastructure			
3	Alp_tour	Touristic alpine areas			3	Medium level of wind infrastructure			
4	Plat_agri	Agricultural Plateau			4	High level of wind infrastructure			
5	Plat_urb	Urban plateau							
6	Jura	Jura ridges							
7	Alp_urb	Urban alpine valley							
									

Attribute Photovoltaic infrastructure					Attribute Power line				

1	No PV infrastructure				1	Absence of high voltage overhead power lines			
2	Low level of PV infrastructure				2	Presence of high voltage overhead power lines			
3	Medium level of PV infrastructure								
4	High level of PV infrastructure								

For reasons of confidentiality we anonymized the data by removing all fields that would enable personal identification. The complete questionnaire, the dataset and data description are available on the Environmental Data Platform EnviDat of the Swiss Federal Institute for Forest, Snow and Landscape Research WSL (https://doi.org/10.16904/envidat.206).

## 2 Experimental Design, Materials and Methods

The representative online panel survey was open for response from November 2018 to March 2019. Within this time, two reminders were sent. The survey targeted active Swiss panel members of panel operator BILENDI. In five months of operation we received a total of 1026 responses. We administered the online questionnaire with the hosting service provided by Sawtooth, while respondents were provided by panel operator BILENDI GmbH. For the layout of the questionnaire we used Sawtooth's survey software Lighthouse Studio [3]. Data cleaning due to item-nonresponse led to a total number of 844 respondents (12,660 choice observations).

The questionnaire consisted of two main parts. The first part consisted of item-based questions regarding landscape and renewable energy infrastructure related aspects. The second part contained a stated choice experiment with fifteen consecutive choice tasks.

### 2.1 The item-based part

The first part of the questionnaire included questions regarding meanings ascribed to landscapes and renewable energy infrastructure, questions related to aspects of landscape-technology fit and questions examining the exposure of people to landscapes and renewable energy infrastructures. All items are presented in Table 1. Item description of items regarding landscape meanings, meanings ascribed to renewable energy infrastructure and landscape-technology fit are presented in Table 2, Table 3 and Table 4.

### 2.2 The choice experiment part

The choice experiment consisted of fifteen consecutive choice tasks. Ich each choice task respondents had to choose between three alternatives. Option 1 and 2 described mixed landscape related renewable energy scenarios (action), whereas option 3 described an opt-out (no-action). Relevant attributes and credible attribute levels were developed based literature research, project meetings and workshops with the project steering group from different disciplines We identified four relevant attributes and the respective levels. The choice design was generated with Ngene software [4] and was designed as d-efficient design that varies the attribute levels in Options 1 and 2. Attribute, attribute levels and the generated choice design are presented in Table 5. A detailed description of the attribute levels and the choice experiment can be found in the accompanying publication [2].

## Ethics Statement

The participation in the survey was operated and organized by a panel provider. Respondent participation was voluntary and respondents were informed that the data will be analyzed anonymously. Data collection and handling were implemented in accordance with the social data gathering ethics regulations of the institution conducting this research.

## CRediT Author Statement

**Salak B.:** Resources, Methodology, Conceptualization, Formal analysis, Investigation, Data Curation, Visualization, Writing - original draft; **Lindberg K.:** Methodology, Formal analysis, Writing - review & editing, Software, Validation; **Kienast F.:** Funding acquisition, Conceptualization, Writing - review & editing, Validation; **Hunziker M.:** Funding acquisition, Project administration, Conceptualization, Writing - review & editing, Validation, Supervision.

## Declaration of competing interest

The authors declare that they have no known competing financial interests or personal relationships which have or could be perceived to have influenced the work reported in this article.

## Appendix Supplementary materials

Image, application 1
