# Supplementary material for: Hybrid choice model dataset of a representative Swiss online panel survey on peoples’ preferences related to mixed renewable energy scenarios in landscapes and the effect of landscape-technology fit
Source: Data Brief. 2021 Apr 16;36:107025. doi: 10.1016/j.dib.2021.107025 (PMC8131564; doi:10.1016/j.dib.2021.107025)
Supplement: Supplementary file 1 [file mmc1.pdf]

Questionnaire related to hybrid choice model

**Language region** From which Swiss region do you come from?	Swiss-German	Swiss-French	Swiss-Italian
	☐	☐	☐

**Gender** Please indicate your gender.	male	female
	☐	☐

**Age** To which of the following age categories do you belong?	18-24	25-34	35-44	45-54	55-64
	☐	☐	☐	☐	☐

**Education** What is the level of your education status?	obligatory school	secondary level: professional education	secondary level: general education	tertiary level: professional education	tertiary level: universities
	☐	☐	☐	☐	☐

LS *Hidden question: Respondents were assigned to one of seven landscapes according to the ZIP codes of their living environment.*

Landscape 1: Near natural alpine areas

Landscape 2: Northern Alps

Landscape 3: Touristic alpine alps

Landscape 4: Agricultural plateau

Landscape 5: Urban plateau

Landscape 6: Jura ridges

Landscape 7: Urban alpine valley

WumgSEL Which of the following landscape types represent your living environment most?

Note: All items were randomized in sequence.

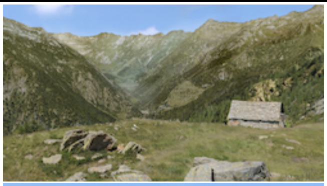 ☐

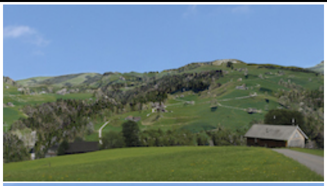 ☐

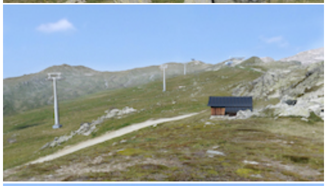 ☐

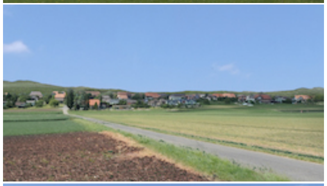 ☐

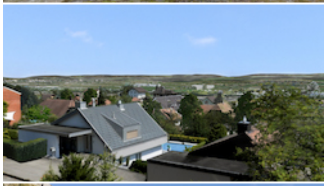 ☐

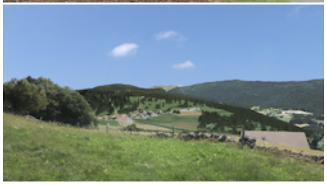 ☐

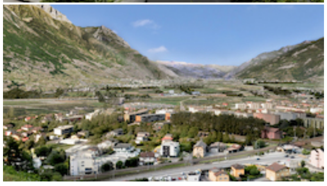 ☐

Questionnaire related to hybrid choice model

LumgSEL Which of the following typical Swiss landscapes best represents your favorite landscape?

Note: All items were randomized in sequence.

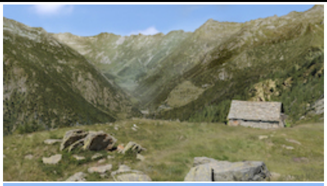 ☐

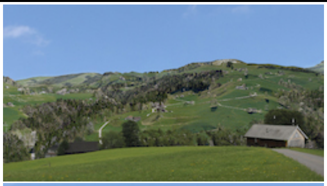 ☐

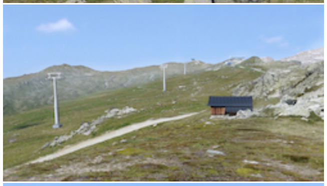 ☐

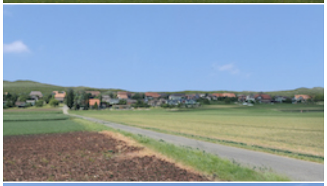 ☐

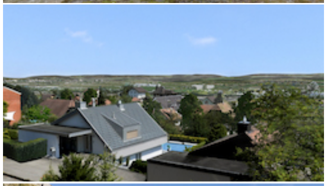 ☐

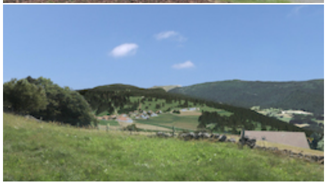 ☐

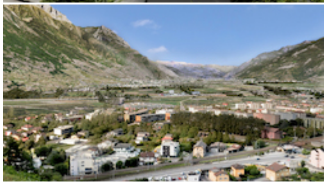 ☐

WgeschKID Which of the following typical Swiss landscapes <u>most closely</u> represents **the landscape of your childhood**?

Note: All items were randomized in sequence.

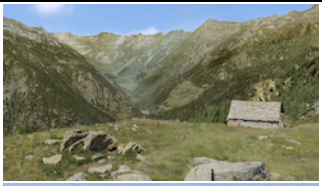 ☐

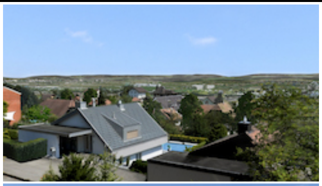 ☐

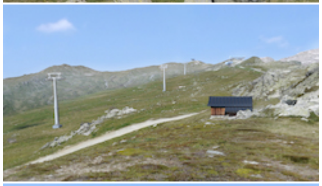 ☐

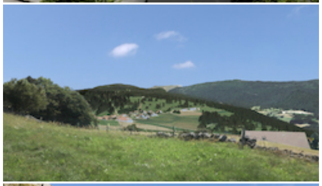 ☐

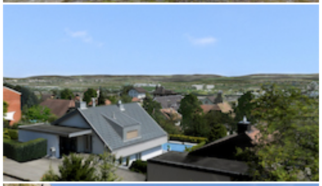 ☐

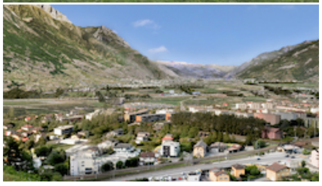 ☐

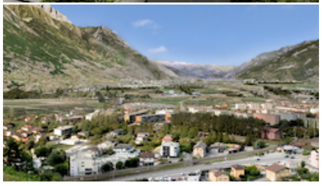 ☐

Questionnaire related to hybrid choice model

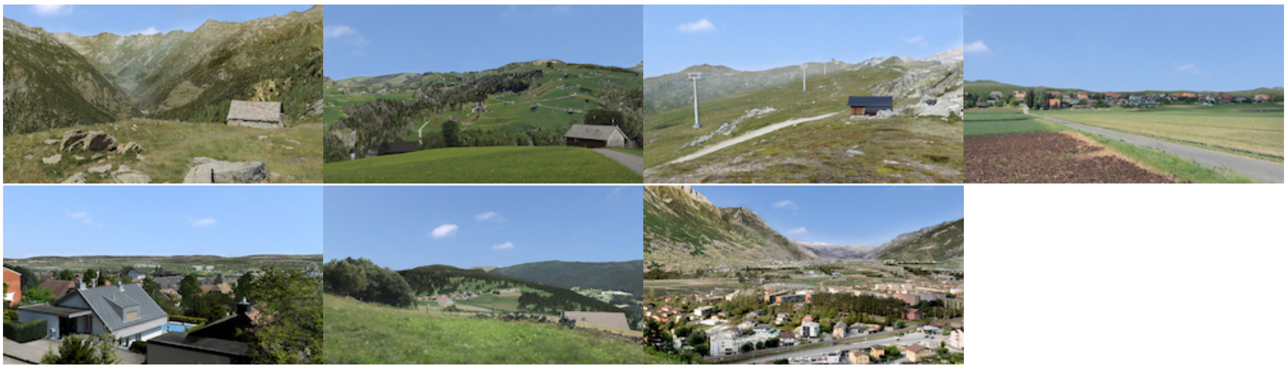

meanings **In your opinion**, do the following statements apply or not apply to the landscape depicted above?

Note: These items were presented randomized and separately for each of the seven landscapes. Only one landscape was visually presented at the same time without any further description. All items were randomized in sequence.

**This Landscape...**	strongly disagree	disagree	in between	agree	strongly agree
...is a symbol for human progress	☐	☐	☐	☐	☐
...represents the harmony between humans and nature	☐	☐	☐	☐	☐
...represents the dominance of humans over nature	☐	☐	☐	☐	☐
...represents the threat to nature	☐	☐	☐	☐	☐
...represents scenic beauty	☐	☐	☐	☐	☐
...offers sense of intimacy/familiarity	☐	☐	☐	☐	☐
...helps to recognize sense	☐	☐	☐	☐	☐
...makes me feel as a part of the region	☐	☐	☐	☐	☐
...helps to can relax my soul	☐	☐	☐	☐	☐
...makes me feeling comfortable	☐	☐	☐	☐	☐
...is a symbol for an authentic landscape	☐	☐	☐	☐	☐
...represents an intact world	☐	☐	☐	☐	☐
...helps to experience myself	☐	☐	☐	☐	☐
...helps to feel safe	☐	☐	☐	☐	☐
...offers possibilities for self-experience	☐	☐	☐	☐	☐
...represents swiss identity	☐	☐	☐	☐	☐
...function oriented landscape	☐	☐	☐	☐	☐
...typical landscape	☐	☐	☐	☐	☐
...unique landscape	☐	☐	☐	☐	☐
...regular landscape	☐	☐	☐	☐	☐

Questionnaire related to hybrid choice model

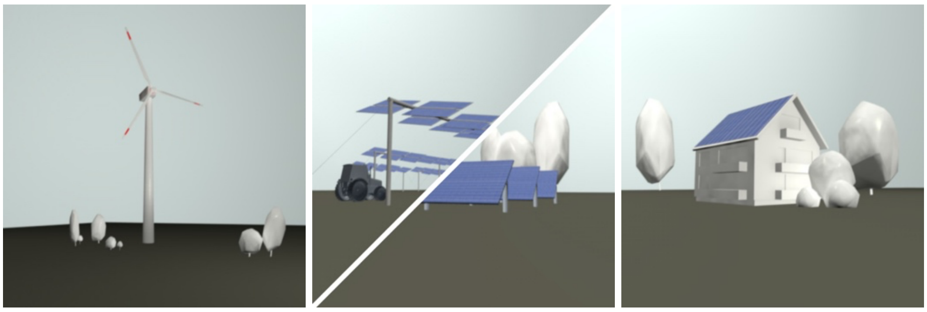

RE **In your opinion**, do the following statements apply or not apply?

Note: These items were presented randomized and separately for each energy infrastructure (Wind, PV-Ground and PV-roof). Only one energy infrastructure was visually presented at the same time without any further description. All items were randomized in sequence.

**Wind Energy...**	strongly disagree	disagree	in between	agree	strongly agree
...provides clean energy	☐	☐	☐	☐	☐
...secures jobs	☐	☐	☐	☐	☐
...supports local economy	☐	☐	☐	☐	☐
...cannot replace other energy sources in CH	☐	☐	☐	☐	☐
...deliver limited yield	☐	☐	☐	☐	☐
...only business for some	☐	☐	☐	☐	☐
...ensures variety in the landscape	☐	☐	☐	☐	☐
...should be developed wherever possible	☐	☐	☐	☐	☐
...represent the progress of humans	☐	☐	☐	☐	☐
...are in harmony with nature	☐	☐	☐	☐	☐
...mechanization of the landscape	☐	☐	☐	☐	☐
...contribute to solving the most important problems of humanity	☐	☐	☐	☐	☐
...represent awakening	☐	☐	☐	☐	☐
...represent feasibility obsession of humans	☐	☐	☐	☐	☐
...distract from really important measures	☐	☐	☐	☐	☐

PTF **In your opinion**, how do you think the following energy infrastructures fit with these landscapes?

Note: This question was asked for Landscapes 1-7. Only the interactions of the energy infrastructure with the landscape 1 are exemplified (PTFaband_r1-5). All items were randomized in sequence per landscape.

	very poor	poor	fair	good	very good
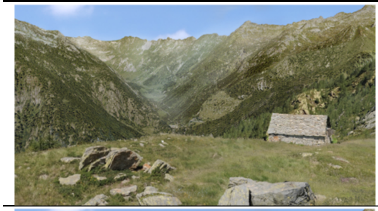 + 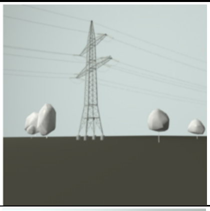	☐	☐	☐	☐	☐
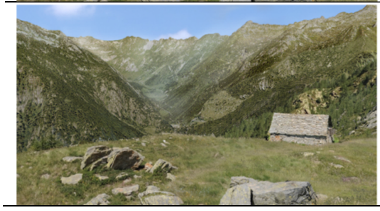 + 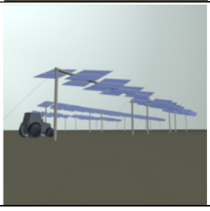	☐	☐	☐	☐	☐

Questionnaire related to hybrid choice model

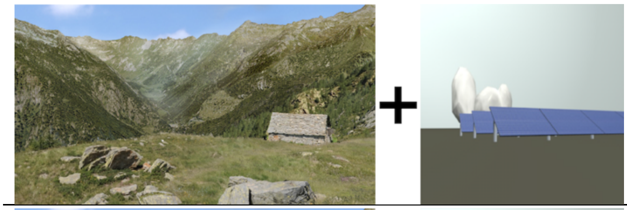	☐	☐	☐	☐	☐
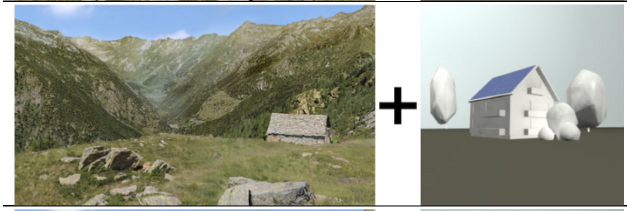	☐	☐	☐	☐	☐
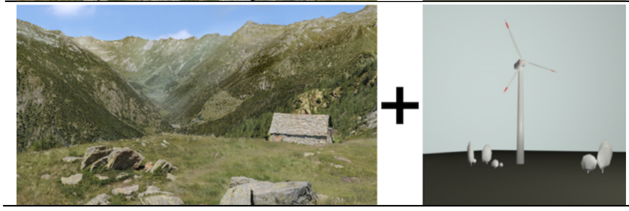	☐	☐	☐	☐	☐

WBTR How do you feel about these energy installations in your living environment?

Note: This question was also asked about one's individual recreation environment (LBTR).

	are very disturbing	are disturbing	rather disturb	neither	rather like	like	like it very much
I feel … about wind energy infrastructures	☐	☐	☐	☐	☐	☐	☐
I feel … about roof mounted PV	☐	☐	☐	☐	☐	☐	☐
I feel … about open space mounted PV	☐	☐	☐	☐	☐	☐	☐

Choice Experiment If you had to decide between the following landscape developments as part of the energy transformation, what would be your decision?

Note: This question was asked for 15 choice tasks. Choice tasks were presented in randomized sequence.

**Scenario 1**	**Scenario 2**	
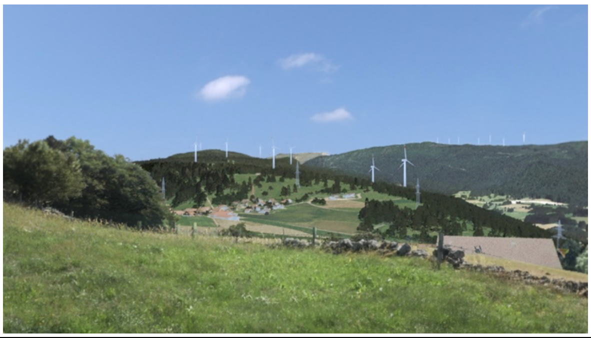	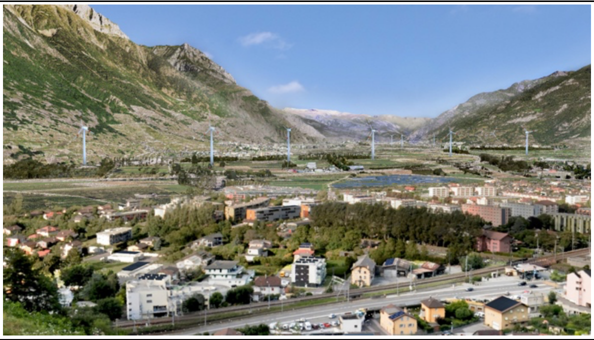	NONE: I cannot decide
Select	Select	Select
